# Longitudinal data collection in pediatric and adult patients with 5q spinal muscular atrophy in Latin America: LATAM RegistrAME study - a clinical registry study protocol

**DOI:** 10.31744/einstein_journal/2024AE1133

**Published:** 2024-11-12

**Authors:** Elice Carneiro Batista, Edmar Zanoteli, Frederico Monfardini, Gustavo Prado dos Santos, Gisele Sampaio Silva, Otávio Berwanger, Luiz Vicente Rizzo, Henrique Andrade Rodrigues da Fonseca

**Affiliations:** 1 Hospital Israelita Albert Einstein São Paulo SP Brazil Hospital Israelita Albert Einstein, São Paulo, SP, Brazil.; 2 Department of Neurology Faculdade de Medicina Universidade de São Paulo São Paulo SP Brazil Department of Neurology, Faculdade de Medicina, Universidade de São Paulo, São Paulo, SP, Brazil.; 3 Imperial College London London United Kingdom Imperial College London, London, United Kingdom.; 4 George Institute for Global Health London United Kingdom George Institute for Global Health, London, United Kingdom.

**Keywords:** Muscular atrophy, spine, 5q-SMA, Rare disease, Motor milestone

## Abstract

Spinal muscular atrophy is a rare hereditary neurodegenerative disease characterized by progressive motor neuron loss. The most common form of SMA is linked to 5q (5q-SMA) and is classified into subtypes according to the age of onset and maximum motor function achieved. The severity ranges from progressive infantile paralysis and premature death (type 1) to limited motor neuron loss in adults (type 4). The LATAM RegistrAME protocol is an observational, retrospective, prospective international multicenter clinical registry study involving Latin Americans. We will include 5q-SMA patients across all age groups, from pediatric to adult, to describe their clinical characteristics and disease progression. The protocol will use an international clinical research form based on the Treat Neuromuscular Disease Global Academic Network (TREAT-NMD), which focuses on advancing research on neuromuscular disorders in patients with spinal muscular atrophy. The study will include six countries in Latin America, sampling a total of 415 patients with SMA and 5q-SMA types 1, 2, 3, and 4 of all ages and both sexes who have genetically confirmed 5q-SMA. The mean follow-up period was 24 months. The primary outcomes will be the description of general clinical characteristics, access to medications capable of modifying the course of the disease, functional assessments, motor milestones, and ventilatory support. This study will provide insights into 5q spinal muscular atrophy patients in Latin America to better understand their clinical characteristics, use of medications, and outcomes while supporting further clinical management protocols and clinical trials. ClinicalTrials.gov Identifier: NCT05475691.

## In Brief

The 5q spinal muscular atrophy (5q-SMA) is a rare hereditary neurodegenerative disease characterized by progressive motor neuron loss. Data on disease progression, medication access, and functional assessments are scarce in Latin America. Batista et al. proposed the first international prospective clinical registry study of 5qSMA patients in Latin America, the LATAM RegistrAME study.

## Highlights

■ LATAM RegistrAME is the first clinical registry study of spinal muscular atrophy linked to 5q-SMA in Latin America (LATAM), including all ages.

■ This study will provide the first clinical evidence of access to medication for 5q-SMA treatment in LATAM.

■ It will describe the functional assessment and ventilatory support of 5q-SMA patients with diverse sociodemographic aspects.

## Graphical abstract

**Figure:**
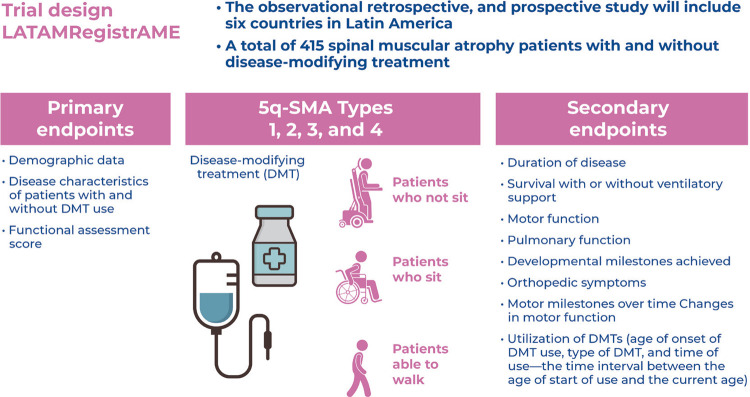

## INTRODUCTION

Spinal muscular atrophy (SMA) is a neuromuscular genetic disorder characterized by progressive anterior horn motor neuron loss in the spinal cord and subsequent progressive weakness and disability.^(1)^ The most common form of SMA, 5q-SMA, is an autosomal recessive disorder caused by homozygous pathogenic variants of the survival motor neuron 1 (*SMN1*) gene on chromosome 5q13.2, with an incidence of 1:6,000-1:10,000.^(2,3)^ Before the emergence of treatments modifying the natural history of the disease, 5q-SMA was considered one of the most devastating childhood neurological diseases. It was the number one cause of death related to monogenetic dysfunction in children.^(1,4)^ The disease severity is highly variable and correlates with the age of onset and maximum motor function achieved, according to which 5q-SMA is classified into four subtypes. Type 1 SMA (the most severe form) presents with onset within the first 6 months of life, with affected children unable to sit unaided.^(1-5)^ Type 2 SMA (intermediate form) manifests between 6 and 18 months of age, with affected children unable to stand or walk unaided. Type 3 SMA (a mild form) typically emerges after the second year of life, with affected individuals able to walk unaided.^(1-3)^ An additional mild adult form, known as type 4 disease, presents with a slow progression of weakness.^(5,6)^

A consensus statement on the standards of care in the 5q-SMA was published in 2017.^(4,7)^ Disease management is mainly based on multidisciplinary management to improve respiratory, gastrointestinal, and orthopedic symptoms. With technical advancements (and thus the possibility of providing noninvasive or invasive ventilatory and nutritional support to affected patients), survival has increased. However, the standards of care provided to patients with 5q-SMA vary significantly.

Recent advances in understanding 5q-SMA pathogenesis have prompted the emergence of promising therapeutic strategies, and several clinical trials have been performed worldwide.^(7-12)^ Nusinersen, an antisense oligonucleotide, was the first disease-modifying treatment (DMT) approved for 5q-SMA treatment in Brazil (08/2017), Argentina (03/2019), Chile (01/2018), Colombia (04/2019), Mexico (11/2018), and Uruguay (07/2018). Gene replacement therapy (onasemnogene abeparvovec) and small-molecule drugs (risdiplam) have also been approved or are awaiting approval, in these countries.

Improvements in treatment and technological advances have changed 5q-SMA’s accessibility of diagnosis and therapy. The systematic collection of data from routine clinical practices in multiple Latin American (LATAM) countries, harmonized with an internationally aligned core dataset, is essential for understanding the natural history of disease in the region and the influence of different drug treatments on patient outcomes. Such data are critical for improving patient care. Clinical trials regarding therapeutic approaches for patients with 5q-SMA have covered only a subgroup of the broad spectrum of 5q-SMA severity. However, numerous 5q-SMA patients still lack access to DMT. Thus, there is an urgent need to monitor the full range of patients treated and untreated with 5q-SMA in a real-world context.

The LATAM RegistrAME study will include a regional healthcare provider (HCP) entered into the registry. The planned 5q-SMA registry will provide an online platform for collecting longitudinal data on 5q-SMA patients in the LATAM to enhance our understanding of patients’ clinical characteristics, disease progression, use of DMTs, and patient outcomes, and facilitate additional research projects and regional data generation.

Data will be collected to make it possible to analyze the clinical evolution of patients over time. The main data sets will be demographic data, characterization of motor condition (motor milestones achieved and also motor milestones lost throughout the study), contractures, motor function, and motor scale scores used in routine clinical care. Information will also be collected on medications capable of altering the disease course, use of a feeding tube, need for ventilatory support, type of ventilatory support, presence of scoliosis, and need and number of hospitalizations.

## OBJECTIVE

The LATAM RegistrAME aims to systematically record and characterize the progression of patients with 5q-SMA during the data collection period to provide insights into the natural progression of the disease over time and observe the effects of medication on the disease course.

## METHODS

### Study design

The LATAM RegistrAME study will be an investigator-initiated, retrospective, prospective, and multicenter nonrandomized registry of LATAM. The variables included in the LATAM RegistrAME registry were based on the core items defined by the TREAT-NMD for 5q-SMA registries and the LATAM RegistrAME steering committee consensus (Table 1S, Supplementary Material). Demographic characteristics, dates of genetic test results, clinical diagnosis, functional status, and pulmonary function were included in the LATAM RegistrAME study.

The LATAM RegistrAME study will include retrospective clinical data from participating centers and offer a standardized structure for prospective data collection. For these cases, it is anticipated that centers in LATAM will be included in planned regular registry-related investigations. The study steering committee will select these reference centers based on their potential to enroll patients and conduct appropriate follow-ups and their experience in treating 5q-SMA and conducting clinical trials. Table 2S, Supplementary Material describes the principal investigators and participating LATAM centers.

### Study population

A complete list of the inclusion and exclusion criteria is provided in table 1. Briefly, we will include approximately 415 LATAM participants with genetically confirmed 5q-SMA types 1, 2, 3, and 4 of all ages and sexes who provided consent to participate in the study. The patients invited to participate in the study underwent health monitoring in institutions with infrastructure and a multidisciplinary team familiar with the care of 5q-SMA patients and the administration of motor functional scales. Participants may or may not use disease-modifying therapies.

**Table 1:** Study enrolment criteria

Inclusion criteria
1. Genetically confirmed 5q-SMA patients at all ages
2. Consent to participate in the study, expressed by the patient or responsible or legal guardian of the pediatric patient/ responsible or legal guardian of the patient with cognitive impairment of understanding the registration protocol
**Exclusion criteria**
1. Patients without a genetic diagnosis confirming 5q-SMA
2. Other types of SMA (non 5q-SMA)
3. Patients who do not accept to participate in the observational study
4. Patients without the legal capacity, who are unable to understand the nature, significance, and consequences of participating in the registry, or, in such cases, without a legal or responsible guardian

### Study procedures

**Figure 1:**
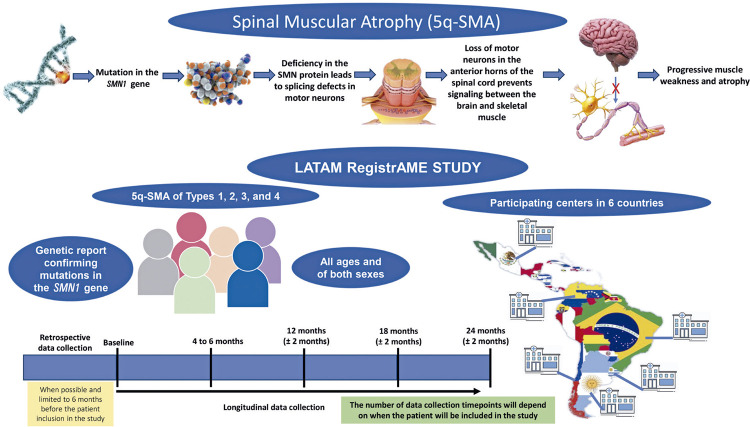
Trial design of LATAM RegistrAME study

At screening, medical records will begin after confirmation of eligibility and informed consent, as well as data collection on health conditions, treatments performed, and clinical evolution. Retrospective data collection (limited to 6 months before patient inclusion in the study), baseline data, and longitudinal data collection will commence. Data entry will scheduled at 4-6 month intervals, depending on the regular healthcare planning of each clinical site (Figure 1).

SMN1: survival motor neuron 1.

All data will be entered into the web-based e-CRF system in REDCap. The principal investigator from each participating center will assign a data entry person to their center. Access to the electronic form for data collection (e-CRF) will be provided by ARO Einstein when the participating centers complete training to participate in the study.

Data for the registry will be obtained through regular, clinically recommended routine visits of patients with 5q-SMA, depending on the current treatment regimen at each center. One person from each participating center will be responsible for entering data into the eCRF; they should be trained physicians, physiotherapists, nurses, or other persons designated by the hospital (site coordinator) to ensure data entry quality.

Participants will undergo follow-up for up to 24 months. The number of data collection time points depends on when the patient is included in the study (with an anticipated minimum of three longitudinal data collections per patient). Data collection from each patient will be carried out approximately every six months within the clinical routine of the centers that monitor patients (real-world context).

### Study endpoints

#### Primary outcome

The co-primary endpoints were demographic data, disease characteristics of patients with and without DMT, and functional assessment score range.

The functional assessment score range will be evaluated using the Children’s Hospital of Philadelphia Infant Test of Neuromuscular (CHOP-INTEND), Hammersmith Infant Neurological Examination Part 2 (HINE-2), Hammersmith Functional Motor Scale Expanded (HFMSE), Revised Upper Limb Module (RULM), and 6 Minute Walk Test (6 MWT).

#### Secondary endpoints

We will analyze data related to disease duration, survival with or without ventilatory support, motor function, pulmonary function, developmental milestones achieved, growth parameters, and orthopedic symptoms. This study will also evaluate ventilation with or without ventilatory support, scoliosis diagnosis and progression, survival with or without ventilatory support, and motor function (ability to sit, sit without support, walk without support, stand without support, and walk independently). The following secondary outcomes will be analyzed by 5q-SMA type, DMT use, and other characteristics, such as disease duration or functional ability to account for disease heterogeneity: disease characteristics at first diagnosis (early signs and symptoms leading to the clinical diagnosis of 5q-SMA); disease duration (time interval between the age at which the first signs and symptoms appeared and the current age); time from 5q-SMA symptom onset until genetic diagnosis; motor milestones over time; changes in motor and pulmonary function, developmental milestones, growth parameters, orthopedic symptoms, scoliosis diagnosis, and Cobb angle (progression of scoliosis over the study duration) will observed throughout the study, and utilization of DMTs (age at onset of DMT use, type of DMT, and time of use; the time interval between the start of use and the current age).

#### Sample size and statistical considerations

As 5q-SMA is a rare disease, this study will use an opportunity sample. Based on the available current epidemiological data within LATAM and information on the number of patients seen at the potential centers in this registry, the estimated registration number was approximately 415 participants. No restrictions will be applied on age, sex, 5q-SMA type, or other characteristics. The statistical analysis will be mainly descriptive because it is a longitudinal data record will use to monitor the full range of treated and untreated 5q-SMA patients in a real-world context. The aim will be to characterize and describe the evolution of the patient’s condition through data collection from the registry and to describe the natural history of the disease in a real-life context.

## Statistical analysis

Baseline characteristics will summarized according to the type of 5q-SMA. Mean and standard deviation will be used for continuous measurements. Categorical variables will described by the proportion of each category (with the corresponding sample sizes). Longitudinal data collection will be conducted at intervals of approximately six months (according to the type of 5q-SMA and the care routine for patients in the health service). Therefore, longitudinal data will ideally be collected at 6, 12, and 18 months after patient inclusion. The reference date for determining data update intervals in the electronic clinical records of the study will be the date of consent to participate. For patients treated with DMTs, the demographic data and disease characteristics at the time of DMT initiation will be descriptively summarized and evaluated. The effectiveness outcomes will be outlined, including survival time and rate (with or without ventilatory support) and changes in motor function, pulmonary function, developmental milestones, growth parameters, and orthopedic symptoms after DMT initiation. If the sample size and data permit, changes in these outcomes may be compared to natural history of untreated patients in the registry or natural history of patients before DMT initiation.

Patient characteristics (*i.e*., demographic data and disease characteristics at first diagnosis) will be displayed. Continuous data will be summarized as the arithmetic mean, standard deviation, minimum, 1^st^ quartile, median, 3^rd^ quartile, maximum, and complete and missing observations. If appropriate, the continuous variables can be presented as categories. Categorical data (such as safety data) are summarized as the total number of patients in each category and the number of missing values. Relative frequencies will be displayed as valid percentages (number of patients divided by the number of patients with non-missing values).

Landmarks such as the onset of the first symptoms, proof by genetic testing, and initiation of disease-modifying treatment will be used to describe the patient’s journey. These will be presented in density graphs and stratified by SMA type and country.

The impact of the intervention on the natural evolution of the disease at specific time points, such as 6, 12, and 18 months after treatment initiation, will be evaluated according to 5q-SMA type, DMT type, and other characteristics, such as disease duration or functional ability. A significance level of 5% will be used, and corrections for multiple tests, such as the Bonferroni correction, may be applied.

## Ethics

This study was approved by the Research Ethics Committee of the *Hospital Israelita Albert Einstein* (CAAE: 58747822.9.1001.0071; #5.585.680).

The local ethics committees of the participating centers in LATAM countries approved the protocol.

### Trial coordination and data management

**Figure 2:**
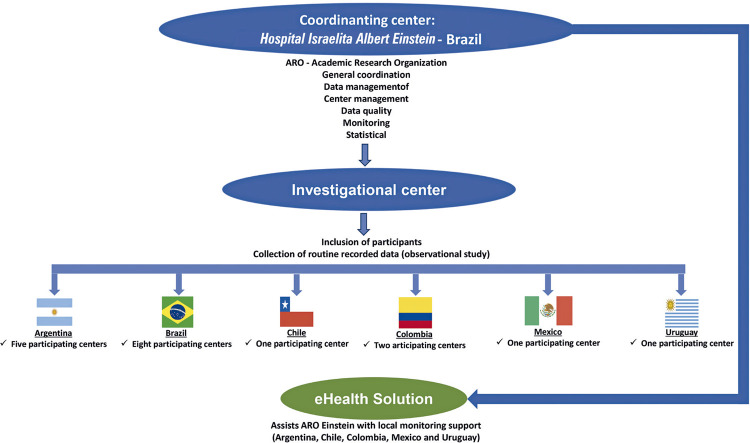
Interaction between the coordinating center and participating centers in LATAM RegistrAME study

The study coordination center will be ARO-Einstein at *Hospital Israelita Albert Einstein*, Sao Paulo-SP, Brazil (Figure 2).

### Data quality and center management

The data will be entered into the web-based e-CRF system from REDCap^®^. The data collection and management system described above has been validated in previous studies and is safe and reliable. The system functionalities include patient registration, data entry, data cleaning, and export for statistical analysis.

The data management team at ARO-Einstein has access to data from all the centers to perform curation, pseudo-anonymization, and data monitoring. Before starting data entry, information on training will be provided via teleconference, supported by digital material. Further support may be provided via teleconference while maintaining the quality of the collected data. The data will be anonymized and encrypted, and all protection and security measures regarding the collection, storage, processing, and sharing of data will be implemented following the local recommendations of the data protection law.

The collection and use of data will adhere to the guidelines outlined in the consent form signed by either the patient or the party responsible for the patient. Data will be anonymized, and each patient will be identified in the registry using a unique patient identification code (patient number). A system to prevent duplicate patient entries will be implemented, and each center will maintain a subject identification log with the names of all registry patients and the corresponding identification codes assigned to each patient. Data security will be addressed according to the local regulations in each participating center.

Several procedures will guarantee the quality of the data, including: all researchers will participate in a training session before the start of the study to ensure consistency in study procedures, including data collection; researchers will have the option to call the study’s coordinating center to resolve any issues or problems that may arise; data entry through the data management system is subject to several checks for open fields; plausible, possible, and disallowed ranges of values; and logical checks. The investigator who enters the data will be notified of any problems during data entry; statistical techniques to identify inconsistencies will be conducted periodically (approximately every 15 days). The centers will be notified of inconsistencies so that they can provide corrections; statistical techniques for identifying data inconsistencies \ will be conducted periodically (every 90 days); monitoring at the centers will be conducted during the study period, and the coordinating center will reviews the detailed monthly reports on screening, inclusion, follow-up, consistencies, and data completeness. It will immediately take action to resolve problems.

## DISCUSSION

The clinical presentation of 5q-SMA varies widely, and the patient’s journey to receive a diagnosis and access treatment, including DMT, is often complex and time-consuming. The progressive and degenerative nature of the disease, primarily due to the denervation process, significantly affects patients’ quality of life. It is crucial to note that individualized treatment plans are based on patient age, disease severity, and response to therapy. Regular monitoring and evaluation are vital to optimize treatment outcomes in patients with 5q-SMA.^(13)^ In a previous cross-sectional study of a Brazilian cohort of patients with 5q-SMA types 2 and 3, as registered in the study NCT04404764,^(14)^ we observed that the interval from the first signs and symptoms to obtaining a confirmatory genetic diagnosis of 5q-SMA could span years. This delay is associated with clinical deterioration, which complicates the evaluation of clinical improvements after treatment. To overcome the limitations of the cross-sectional approach, the LATAM RegistrAME study aimed to conduct a longitudinal follow-up of patients. These follow-ups will expand beyond Brazil to cover a broader spectrum of patients with LATAM and will not be limited to types 2 and 3 of the disease.

In the last few years, the advent of DMT has dramatically changed the 5q-SMA disease course, with an increasing number of patients showing improvements or at least stabilization in motor and respiratory functions. Changes in disease progression have led to unexpected results, such as sitting in type 1 patients and walking in type 2 patients.^(15,16)^ The management of 5q-SMA is profoundly changing, and several issues must be considered as clinicians use these new, innovative, and effective treatments. Understanding the clinical profile of patients with LATAM, the reality of care, and the evolution of conditions over time concerning the treatments administered is necessary. Currently, we lack a robust database of patients in LATAM to facilitate the disease progression analysis in 5q-SMA patients, both with and without access to DMT, in a real-world context, including the disease duration and the treatment initiation timing. These data are critical for enhancing patient care, expediting diagnostic procedures, aiding the planning and management of healthcare resources and treatment access, and supporting further research projects and LATAM data generation.

## CONCLUSION

This study will provide essential insights into 5q-SMA patients in the LATAM to better understand patients’ clinical characteristics, natural history of the disease, use of disease-modifying treatments, and patient outcomes, as well as to support further research projects and regional data generation.

## References

[1] Mercuri E, Bertini E, Iannaccone ST (2012). Childhood spinal muscular atrophy: Controversies and challenges. Lancet Neurol.

[2] Lefebvre S, Burglen L, Reboullet S, Clermont O, Burlet P, Viollet L (1995). Identification and characterization of a spinal muscular atrophy-determining gene. Cell.

[3] Shababi M, Lorson CL, Rudnik-Schoneborn SS (2014). Spinal muscular atrophy: a motor neuron disorder or a multiorgan disease?. J Anat.

[4] Mercuri E, Finkel RS, Muntoni F, Wirth B, Montes J, Mainet M (2018). Diagnosis and management of spinal muscular atrophy, part 1: Recommendations for diagnosis, rehabilitation, orthopedic and nutritional care. Neuromuscul Disord.

[5] Finkel RS, McDermott MP, Kaufmann P, Darras BT, Chung WK, Sproule DM (2014). Observational study of spinal muscular atrophy type I and implications for clinical trials. Neurology.

[6] Piepers S, van den Berg LH, Brugman F, Ruiterkamp-Versteeg M, van Engelen BG (2008). A natural history study of late onset spinal muscular atrophy types 3b and 4. J Neurol.

[7] Finkel RS, Mercuri E, Meyer OH, Simonds AK, Schroth MK, Graham RJ (2018). Diagnosis and management of spinal muscular atrophy, part 2: Pulmonary and acute care; medications, supplements and immunizations; other organ systems; and ethics. Neuromuscul Disord.

[8] Finkel RS, Mercuri E, Darras BT, Connolly AM, Kuntz NL, Kirschner J (2017). Nusinersen versus sham control in infantile-onset spinal muscular atrophy. N Engl J Med.

[9] Mendell JR, Al-Zaidy S, Shell R, Arnold D, Rodino-Klapac LR, Prior TW (2017). Single-dose gene-replacement therapy for spinal muscular atrophy. N Engl J Med.

[10] Mercuri E, Darras BT, Chiriboga CA, Day JW, Campbell C, Connolly AM (2018). Nusinersen versus sham control in later-onset spinal muscular atrophy. N Engl J Med.

[11] Verhaart IEC, Robertson A, Leary R, McMacken G, König K, Kirschner J (2017). A multi-source approach to determine SMA incidence and research ready population. J Neurol.

[12] Bladen CL, Thompson R, Jackson JM, Connie Guirlanda C, Wegel C, Ambrosini A (2014). Mapping the differences in care for 5,000 spinal muscular atrophy patients: A survey of 24 national registries in North America, Australasia and Europe. J Neurol.

[13] Zanoteli E, Araujo AP, Becker MM, Fortes CP, França MC, Machado-Costa MC (2024). Consensus from the Brazilian Academy of Neurology for the diagnosis, genetic counseling, and use of disease-modifying therapies in 5q spinal muscular atrophy. Arq. Neuropsiquiatr.

[14] ClinicalTrials.gov NCT04404764: Characterization of the clinical-epidemiological profile of patients with SMA5q types II and III: Observational study.

[15] Messina S, Sframeli M (2020). New treatments in spinal muscular atrophy: Positive results and new challenges. J Clin Med.

[16] Jedrzejowska M, Kostera-Pruszczyk A (2020). Spinal muscular atrophy-New therapies, new challenges. Neurol Neurochir Pol.

