# Supplementary material for: Longitudinal data collection in pediatric and adult patients with 5q spinal muscular atrophy in Latin America: LATAM RegistrAME study - a clinical registry study protocol
Source: Einstein (Sao Paulo). 2024 Nov 12;22:eAE1133. doi: 10.31744/einstein_journal/2024AE1133 (PMC11634359; doi:10.31744/einstein_journal/2024AE1133)
Supplement: Supplementary file 1 [file 2317-6385-eins-22-eAE1133-suppl01.pdf]

SUPPLEMENTARY MATERIAL

Current landscape of clinical use of *ex vivo* expanded Natural Killer cells for cancer therapy

Júlia Teixeira Cottas de Azevedo, Juliana Aparecida Preto de Godoy, Cláudia de Souza, Micheli Severo Sielski, Larissa Leggieri Coa, Augusto Barbosa Júnior, Lucila Nassif Kerbauy, Andrea Tiemi Kondo, Oswaldo Keith Okamoto, Nelson Hamerschlak, José Mauro Kutner, Raquel de Melo Alves Paiva

DOI: 10.31744/einstein_journal/2024RW0612

Table 1S. Currently ongoing clinical trials using natural killer cells expanded via feeder cells

Identification of clinical trails	Title	Phase	Localization	Status	Condition	Dose (cells/kg)	Age (y)	Conditioning regime	Type of feeder cell	Reference
NCT02481934	Clinical Trial using Expanded and Activated Autologous NK Cells to Treat Multiple Myelomas (NK-VS-MM)	I	Hospital Universitario 12 de Octubre	Completed	Multiple myeloma	8 doses : 7.5×10^6 (2 infusions per cycle)	61-72	Lenalidomide or Bortezomib	K562-mb15-41BBL	Leivas et al.[21]
NCT02030561	NK Cell Infusions With Trastuzumab to treat Patients With HER2+ Breast and Gastric Cancers	I/II	National University Hospital, Singapore	Unknown	Breast cancer	1 dose: 1×10^7	32-67	Trastuzumab	K562-mbIL15-41BBL	Lee et al.[6] Yadav et al.[23]
NCT02507154	Reactivating NK Cells in Treating Refractory Head and Neck Cancers (NKEXPHNC)	I/II	National University Hospital, Singapore	Unknown	Carcinoma (nasopharyngeal)	2 doses: 1×10^6 or 1x10^7	45-66	Cetuximab	K562-mbIL15-41BBL	Lim et al.[22]
NCT02123836	Natural Killer Cells in Acute Leukemia and Myelodysplastic Syndrome	I	National University Hospital, Singapore	Unknown	Acute leukemia and myelodysplastic syndrome	1 dose: 1×10^8 (0.5-20×10^7)	6-80	Fludarabine and cyclophosphamide	K562-mbIL15-41BBL	Fujisaki et al.[18]
NCT01974479**	Pilot Study of Redirected Haploidentical Natural Killer Cell Infusions for B-Lineage Acute Lymphoblastic Leukemia	I	National University Hospital, Singapore	Suspended	B-cell acute lymphoblastic leukemia	1 dose: 0,5 ×10^7-1 ×10^8	up to 80	Fludarabine and cyclophosphamide	K562-mbIL15-41BBL	NA
NCT00995137**	Genetically Modified Haploidentical Natural Killer Cell Infusions for B-Lineage Acute Lymphoblastic Leukemia	I	St. Jude Children's Research Hospital	Completed	B-cell acute lymphoblastic leukemia	NA	up to 18	NA	K562-mbIL15-41BBL	NA
NCT02763475	NK Cells as Consolidation Therapy of Acute Myeloid Leukemia in Children/ Adolescents	II	Hospital Infantil Universitario Niño Jesús	Completed	Acute myeloid leukemia	2 doses: 6.92 × 10^6 - 1.93 × 10^8	0.7-16	Fludarabine and cyclophosphamide	K562-mbIL15-41BBL	Gómez García et al.[20]
NCT03209869	Treatment of Relapsed or Refractory Neuroblastoma and Osteosarcoma With Expanded Haploidentical NK Cells and Hu14.18-IL-2	I	University of Wisconsin Carbone Cancer Center	Suspended	Neuroblastoma and Osteosarcoma	NA	0.7-25	NA	K562-mbIL15-41BBL	NA

continue...

...Continuation

Table 1S. Currently ongoing clinical trials using natural killer cells expanded via feeder cells

Identification of clinical trails	Title	Phase	Localization	Status	Condition	Dose (cells/kg)	Age (y)	Conditioning regime	Type of feeder cell	Reference
NCT01944982	Salvage Therapy With Chemotherapy and Natural Killer Cells in Relapsed/ Refractory Pediatric T Cell Lymphoblastic Leukemia and Lymphoma (HNJ-NKAES-2012)	I/II	Hospital Infantil Universitario Niño Jesús	Terminated	Acute T-cell lymphoblastic leukemia	2 doses: first dose up to 5×10^7 and second dose up to 1×10^8	3-19	Nelarabine, etoposide and cyclophosphamide	K562-mbIL15-4-1BBL	Vela et al.[19]
NCT02074657	"LANK-2": Activated and Expanded NK Cell Immunotherapy Together With Salvage Chemotherapy in Children, Adolescents and Young Adults With Relapsed or Refractory Acute Leukemia (LYDIA)	I/II	Hospital Infantil Universitario Niño Jesús	Terminated	Acute myeloid leukemia, T-cell acute lymphoblastic leukemia, B-cell acute lymphoblastic leukemia	4 doses: first and second doses up to 2.5×10^7; third and fourth doses up to 5×10^7	1-23	Clofarabine, etoposide and cyclophosphamide; or Fludarabine, idarubicin, cytarabine and G-CSF; or Fludarabine and cyclophosphamide	K562-mbIL15-4-1BBL	Vela et al.[19]
NCT01287104*	A Phase I Study on NK Cell Infusion Following Allogeneic Peripheral Blood Stem Cell Transplantation From Related or Matched Unrelated Donors in Pediatric Patients With Solid Tumors and Leukemias	I	National Institutes of Health Clinical Center	Completed	Sarcoma	2 doses: 1 ×10^5 or ×x 10^6	4-35	Etoposide, vincristine, adriamycin, prednisone, cyclophosphamide, fludarabine and melphalan	K562-mbIL15-4-1BBL	Shah et al.[46]
NCT02809092	Interleukin-21 (IL-21)-Expanded Natural Killer Cells for Induction of Acute Myeloid Leukemia	I/II	Hospital de Clínicas de Porto Alegre	Unknown	Acute myeloid leukemia	6 doses: 10^6-10^7	2-59	Fludarabine, Cytarabine and G-CSF	K562-mbIL21	Denman et al.[25]
NCT04836390*	Donor-Derived *Ex-Vivo* Expanded Natural Killer Cell Infusions in Children and Young Adults With High Risk Acute Myeloid Leukemia Receiving Myeloablative HLA-Haploidentical Hematopoietic Cell Transplant (EXCEL)	II	Children's Hospital Los Angeles	Recruiting	Acute myeloid leukemia	3 doses: 1×10^8	up to 25	Busulfan and Cyclophosphamide	K562-mbIL21	NA
NCT04220684	Ph1 Trial Test Safety of IL-21 NK Cells for Induction of R/R AML	I	Ohio State University Comprehensive Cancer Center	Recruiting	Acute myeloid leukemia and myelodysplastic syndrome	6 doses: 1×10^7 or 3×10^7 or 1×10^8	18 or more	Fludarabine and Cytarabine or Fludarabine and Decitabine	K562-mbIL21	Vasu et al.[47]
NCT01787474	Donor Natural Killer Cells in Treating Patients With Relapsed or Refractory Acute Myeloid Leukemia	I/II	MD Anderson Cancer Center	Completed	Acute myeloid leukemia and myelodysplastic syndrome	6 doses: 1×10^6 or 5×10^6	12-70	Fludarabine, Cytarabine and G-CSF	K562-mbIL21	Ciurea et al.[26]

continue...

...Continuation

Table 1S. Currently ongoing clinical trials using natural killer cells expanded via feeder cells

Identification of clinical trails	Title	Phase	Localization	Status	Condition	Dose (cells/kg)	Age (y)	Conditioning regime	Type of feeder cell	Reference
NCT03348033	Safety and Feasibility of the Use of Natural Killer Cells in Patients With Chronic Myeloid Leukemia	I/II	Hospital de Clínicas de Porto Alegre	Recruiting	Chronic myeloid leukemia	6 doses	2-59	Fludarabine, Cytarabine and G-CSF	K562-mbIL21	NA
NCT01729091*	Umbilical Cord Blood-Derived Natural Killer Cells, Elotuzumab, Lenalidomide, and High Dose Melphalan, Followed by Stem Cell Transplant to Treat Patients With Multiple Myelomas	II	MD Anderson Cancer Center	Active, not recruiting	Multiple myeloma	1 dose: up to 1×10^8	18-75	Lenalidomide and melphalan	K562-mbIL21	Shah et al.[30]
NCT01904136*	Natural Killer Cells Before and After Donor Stem Cell Transplant to Treat Patients With Acute Myeloid Leukemia, Myelodysplastic Syndrome, or Chronic Myelogenous Leukemia	I/II	MD Anderson Cancer Center	Completed	Acute myeloid leukemia, Chronic myeloid leukemia, Myelodysplastic syndrome	3 doses: 1×10^5 - 1×10^8	18-60	Melphalan, fludarabine and total body irradiation	K562-mbIL21-4-1BBL	Ciurea et al.[26,27]
NCT03056339**	Umbilical & Cord Blood (CB) Derived CAR-Engineered NK Cells for B Lymphoid Malignancies	I/II	MD Anderson Cancer Center	Active, not recruiting	B cell lymphomas	1 dose: 1×10^5 or 1×10^6 or 1×10^7	7-80	Fludarabine and cyclophosphamide	K562-mbIL21-4-1BBL	Liu et al.[48]
NCT04254419	Intra-tumoral Injection of Natural Killer Cells in High-Grade Gliomas (NK HGG)	I	Nationwide Children's Hospital Columbus	Not recruiting	Glioma	NA	3-17	NA	NA	NA
NCT02890758	Phase I Trial of Universal Donor NK Cell Therapy in Combination With ALT803	I	University Hospitals Cleveland Medical Center	Active, not recruiting	Colorectal carcinoma, Myelodysplastic syndrome, Acute myeloid leukemia	2 doses: 1×10^7 or 2.5×10^7 or 5×10^7	18 or more	Fludarabine and cyclophosphamide	NKF cells (OCI/AML3-mbIL21)	Ojo et al.[35] Otegbeye et al.[36]
NCT01212341	Allogeneic Natural Killer (NK) Cell Therapy in Patients With Lymphomas or Solid Tumors (MG4101)	I	Seoul National University Hospital	Complete	Lymphoma or sloid tumor	1 dose: 1×10^6 or 1×10^7 or Repeated doses: 1×10^6 or 3×10^6 or 1×10^7 or 3×10^7	18 or more	NA	PBMC	Yang et al.[38]
UMIN000007527	-	I	Kyoto Prefectural University of Medicine	-	Digestive cancer	3 doses: 0.5 × 109, 1.0 × 109, 2.0 × 109 cells/ injection	48-78	S-1 or none	FN-CH296 induced T cells.	Sakamoto et al.[39]
UMIN000013378	-	I	Kyoto Prefectural University of Medicine	-	Digestive cancer	3 doses: 0.5 × 109, 1.0 × 109, 2.0 × 109 cells/ injection	34-79	capecitabine or S1, cisplatin, Trastuzumab; or capecitabine or S1, oxaliplatin, Cetuximab	FN-CH296 induced T cells.	Sakamoto et al.[39] Ishikawa et al.[40]

* Natural killer cell therapy associated with hematopoietic stem cell transplantation; ** Chimeric Antigen Receptor (CAR)-modified NK cells.
